# A new species and new record of the leafhopper genus *Seriana* Dworakowska (Hemiptera, Cicadellidae, Typhlocybinae) from China

**DOI:** 10.3897/zookeys.172.1741

**Published:** 2012-03-01

**Authors:** Yuehua Song, Zizhong Li

**Affiliations:** 1 Institute of Entomology, Guizhou University, Guiyang, Guizhou 550025, China; 2 Institute of South China Karst, Guizhou Normal University, Guiyang, Guizhou 550001, China; 3 The State Key Laboratory Incubation Base for Karst Mountain Ecology Environment of Guizhou Province, Guiyang, Guizhou 550001, China

**Keywords:** Hemiptera, morphology, taxonomy

## Abstract

*Seriana menglaensis* **sp. n.** (Hemiptera: Cicadellidae: Typhlocybinae: Erythroneurini) is described and illustrated from Southwest China. *Seriana equata* (Singh, 1969) is recorded for the first time from China.

## Introduction

The leafhopper genus *Seriana* was established by Dworakowska (1971) in the tribe Erythroneurini of Typhlocybinae with *Seriana frater* Dworakowska, 1971 as the type species. *Seriana* consists of thirty-three species in the world distributed in Oriental and Palaearctic regions. The genus can be distinguished by the body fuscous, the crown usually with median dark patch on anterior margin of vertex, the pronotum with five oval grey impressed patches near anterior marign; the pygofer hind margin acutely produced, with oblique dorsolateral internal ridge and basolateral setae in distinct group and the pygofer dorsal appendage not movably articulated; the subgenital plate pocket-like apically, with 2–4 basal macrosetae and several short rigid setae on upper margin subbasally; the style apex truncate; the aedeagus usually with pair of processes and the connective nearly Y-shaped.

Only two species, *Seriana indefinita* Dworakowska, 1971 from Guangzhou and *Seriana ochrata* Dworakowska, 1971 from Taiwan were so far reported from China. We describe a new species from Yunnan Province, China and provide illustrations for both the new species and *Seriana equata* (Singh) recorded for the first time from China.

## Methods and materials

The specimens were obtained by sweep net method and were studied under Olympus SZX7 and CX41 microscopes. Morphological techniques and terminology follow Dietrich and Dmitriev (2006). Measurements of the new species are given in millimeters; body length is measured from the apex of the head to the apex of the fore wing in repose. All specimens examined are deposited to the collection of the Insititute of Entomology, Guizhou University, Guiyang, China (GUGC).

## Taxonomy

### Key to species of Seriana from China

**Table:** 

1	Aedeagus with paired processes	2
–	Aedeagus with unpaired processes (Figs 18, 19)	*Seriana indefinita* Dworakowska
2	The paired processes arising from the base of aedeagual shaft (Figs 6, 7)	*Seriana menglaensis* sp. n.
–	The paired processes arising from the midlength of aedeagual shaft	3
3	The paired processes shorter, hook-like (Figs 16, 17)	*Seriana equata* (Singh)
–	The paired processes longer, finger-like (Figs 20, 21)	*Seriana ochrata* Dworakowska

### Seriana menglaensis

Song & Li sp. n.

urn:lsid:zoobank.org:act:80FEEE6B-1EEA-491F-AE03-7EBADD04AF0F

http://species-id.net/wiki/Seriana_menglaensis

Figures 1–10

#### Description.

**Figures 1–10.**
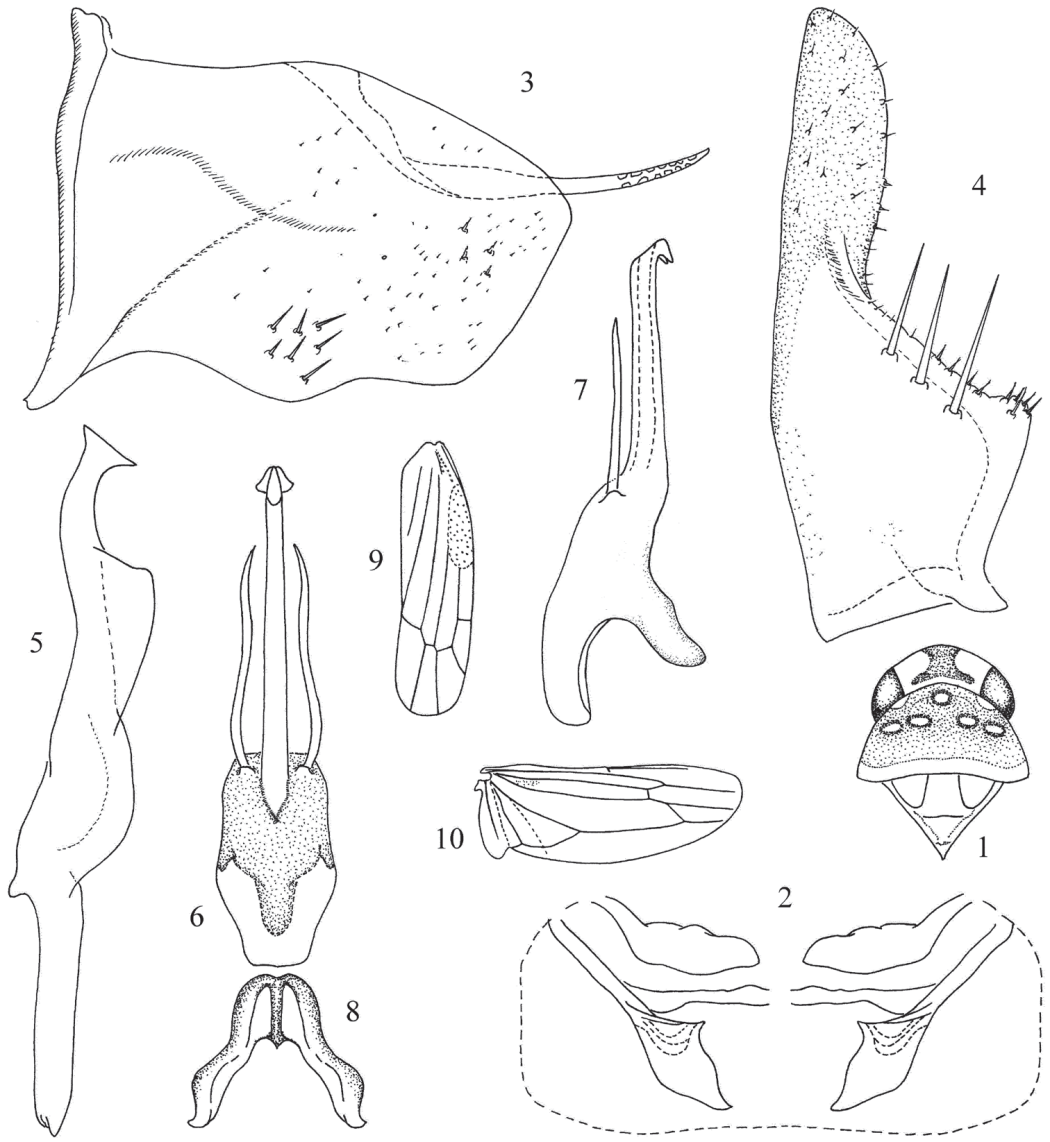
*Seriana menglaensis* Song & Li, sp. n**. (♂) 1** Head and thorax, dorsal view **2** Abdominal apodemes **3** Pygofer lobe, lateral view **4** Subgenital plate **5** Style **6** Aedeagus, ventral view **7** Aedeagus, lateral view **8** Connective **9** Forewing **10** Hind wing.

General color fuscous. Head (Fig. 1) with vertex dirty yellow, with an irregular brown spot at anterior margin medially; eyes black. Pronotum (Fig. 1) with five whitish oval impressed patches near anterior margin. Forewing light testaceous, without markings; brochosome field blackish brown.

Head (Fig. 1) distinctly narrower than pronotum; vertex bluntly rounded.

Abdominal apodemes (Fig. 2) small, acuminate apically, not extended beyond hind margin of 3rd sternite.

Pygofer lobe (Fig. 3) broad, with distinct oblique dorsolateral internal ridge, numerous macrosetae at lower basal angle. Pygofer dorsal appendage very long and fused with dorsal margin of pygofer. Subgenital plate (Fig. 4) with three long macrosetae and short rigid setae at upper margin subbasally; several microsetae scattered on apical portion. Style (Fig. 5) long, apex truncate; preapical lobe large. Connecitve (Fig. 8) Y-shaped, stem strong, central lobe absent. Aedeagus (Figs 6, 7) with shaft long and straight, with two pairs processes, one pair very long, arising from basolateral part of shaft; another pair placed apically very short, lamellate; gonopore at apex, on ventral margin, dorsal apodeme short, weakly expanded.

#### Measurement.

 Body length males 3.2 mm.

#### Type material. 

*Holotype*, male, China: Yunnan Prov., Mengla County, at light, 23 July 2008, coll. Yuehua Song. *Paratype*: one male, same data as holotype.

#### Remarks.

 The new species is similar to *Seriana ochrata* Dworakowska (1971), but can be distinguished from the latter by the aedeagal shaft longer and straighter, similar in width throughout length in ventral view; the paired long processes arising from the base of aedeagal shaft, not at midlength and the dorsal apodeme small.

#### Etymology.

 The new species is named after its type locality: Mengla.

### Seriana equata

(Singh, 1969) rec. n.

http://species-id.net/wiki/Seriana_equata

Figures 11–17

Zygina equata Singh, 1969: 344, figs 20-23
Empoascanara equata (Singh, 1969) (Sohi 1976: 204)
Seriana equata (Singh, 1969) (Dworakowska and Viraktamath 1975: 529, no figures)
Seriana punjabensis Dworakowska, Nagaich & Singh, 1978: 246, figs 38-42 (Syn. by Sohi and Dworakowska 1983: 180)

#### Material examined.

 One male, China: Yunnan Prov., Xishuangbanna, Original Forest Park, 21 July 2008, coll. YUEHUA SONG; one male, China: Henan Prov., Luanchuan, Heyu, 19 August 2008, coll. JIANDA LI.

**Figures 11–21.**
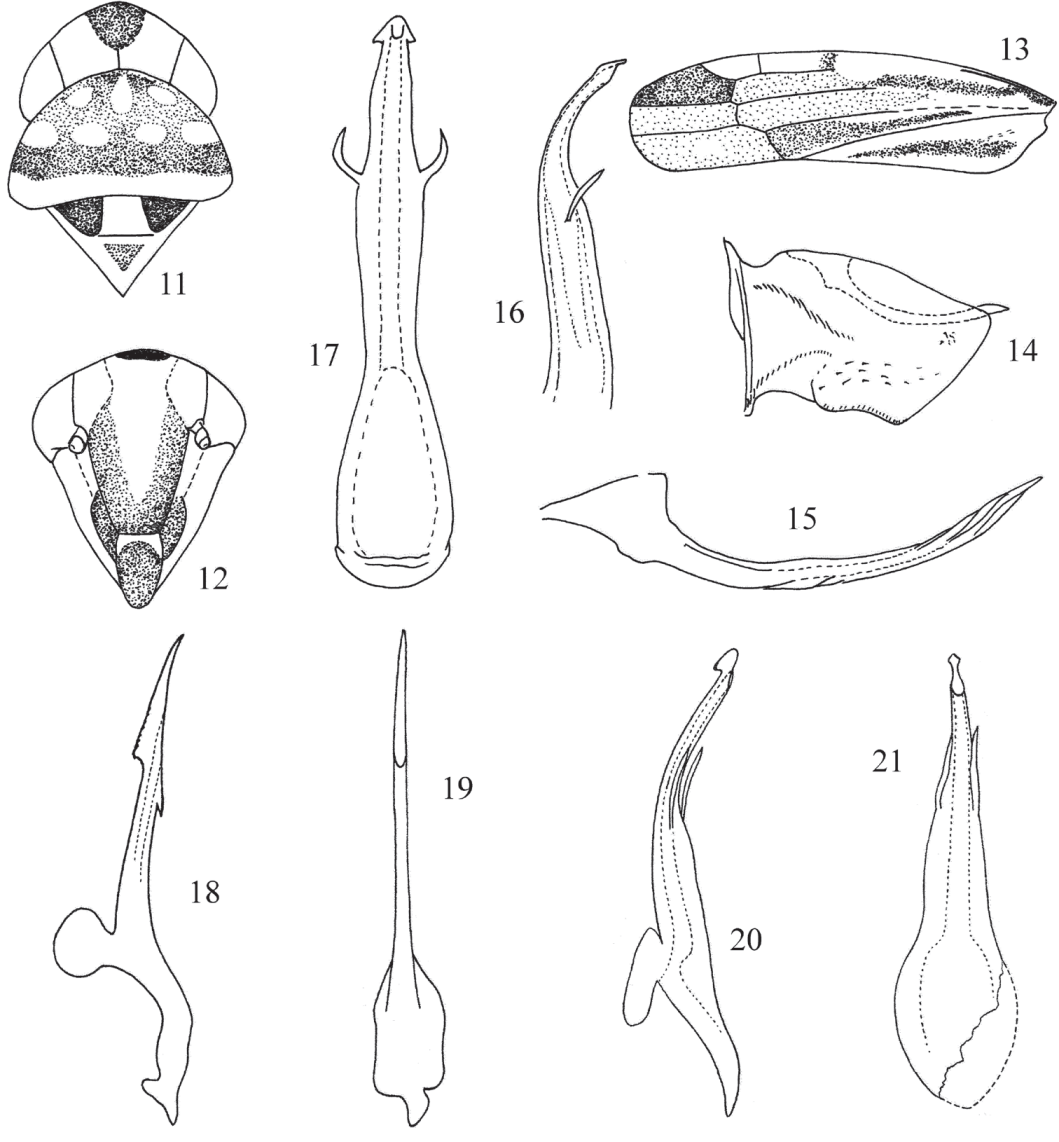
*Seriana equata* (Singh, 1969) rec. n. (after Dworakowska et al. 1978) **11** Head and thorax, dorsal view **12** Face **13** Fore wing **14** Pygofer lobe, lateral view **15** Pygofer dorsal appendage **16** Aedeagus, lateral view **17** Aedeagus, ventral view; *Seriana indefinita* Dworakowska, 1971 **18** Aedeagus, lateral view **19** Aedeagus, ventral view; *Seriana ochrata* Dworakowska, 1971 **20** Aedeagus, lateral view **21** Aedeagus, ventral view. (after Dworakowska 1971)

#### Host plant.

Grasses, potato, black gram, cowpea, Egyptian clover, groundnut, linseed, lucerne, musk melon, spinach, sweet potato, sunnhemp (Sohi and Dworakowska 1983).

#### Distribution.

India; China (Henan, Yunnan).

## Supplementary Material

XML Treatment for Seriana menglaensis

XML Treatment for Seriana equata

## References

[B1] Dietrich CH Dmitriev DA (2006) Review of the New World genera of the leafhopper tribe Erythroneurini (Hemiptera: Cicadellidae: Typhlocycbinae). Illinois Natural History Survey Bulletin 37 (5): 119-190

[B2] Dworakowska I (1971) On some genera of Erythroneurini (Cicadellidae, Typhlocybinae) from the Oriental Region. Bulletin de l’Académie Polonaise des Sciences, Série des Sciences Biologiques 19 (5): 341-350

[B3] Dworakowska I (1976) On some Oriental and Ethiopian Typhlocybinae (Homoptera, Auchenorrhyncha, Cicadellidae). Reichenbachia 16 (1): 1-51

[B4] Dworakowska I Nagaich BB Singh S (1978) *Kapsa simlensis* sp.n. from India and some other Typhlocybinae (Auchenorrhyncha, Cicadellidae). Bulletin de l’Académie Polonaise des Sciences, Série des Sciences Biologiques 26 (4): 243-249

[B5] Dworakowska I Viraktamath CA (1975) On some Typhlocybinae from India (Auchenorrhyncha, Cicadellidae). Bulletin de l’Académie Polonaise des Sciences, Série des Sciences Biologiques 23 (8): 521-530

[B6] Sohi AS Dworakowska I (1983) A review of the indian Typhlocybinae (Homoptera: Cicadellidae) from India. Oriental Insects 17: 159-213

[B7] Sohi AS (1976) New combinations of some Typhlocybines (Homoptera, Cicadellidae, Typhlocybinae) from India. Entomon 1 (2): 203-205

